# An MRPS12 mutation modifies aminoglycoside sensitivity caused by 12S rRNA mutations

**DOI:** 10.3389/fgene.2014.00469

**Published:** 2015-01-14

**Authors:** Sonia Emperador, David Pacheu-Grau, M. Pilar Bayona-Bafaluy, Nuria Garrido-Pérez, Antonio Martín-Navarro, Manuel J. López-Pérez, Julio Montoya, Eduardo Ruiz-Pesini

**Affiliations:** ^1^Departamento de Bioquímica, Biología Molecular y Celular, Universidad de ZaragozaZaragoza, Spain; ^2^Instituto de Investigación Sanitaria de Aragón, Universidad de ZaragozaZaragoza, Spain; ^3^Centros de Investigación Biomédica en Red de Enfermedades Raras, Universidad de ZaragozaZaragoza, Spain; ^4^Fundación ARAID, Universidad de ZaragozaZaragoza, Spain

**Keywords:** mitochondrial ribosomal RNA, pathologic mutation, incomplete penetrance, aminoglycoside, compensatory mutation, mitochondrial ribosomal protein S12, evolutionary approaches

## Abstract

Several homoplasmic pathologic mutations in mitochondrial DNA, such as those causing Leber hereditary optic neuropathy or non-syndromic hearing loss, show incomplete penetrance. Therefore, other elements must modify their pathogenicity. Discovery of these modifying factors is not an easy task because in multifactorial diseases conventional genetic approaches may not always be informative. Here, we have taken an evolutionary approach to unmask putative modifying factors for a particular homoplasmic pathologic mutation causing aminoglycoside-induced and non-syndromic hearing loss, the m.1494C>T transition in the mitochondrial DNA. The mutation is located in the decoding site of the mitochondrial ribosomal RNA. We first looked at mammalian species that had fixed the human pathologic mutation. These mutations are called compensated pathogenic deviations because an organism carrying one must also have another that suppresses the deleterious effect of the first. We found that species from the primate family Cercopithecidae (old world monkeys) harbor the m.1494T allele even if their auditory function is normal. In humans the m.1494T allele increases the susceptibility to aminoglycosides. However, in primary fibroblasts from a Cercopithecidae species, aminoglycosides do not impair cell growth, respiratory complex IV activity and quantity or the mitochondrial protein synthesis. Interestingly, this species also carries a fixed mutation in the mitochondrial ribosomal protein S12. We show that the expression of this variant in a human m.1494T cell line reduces its susceptibility to aminoglycosides. Because several mutations in this human protein have been described, they may possibly explain the absence of pathologic phenotype in some pedigree members with the most frequent pathologic mutations in mitochondrial ribosomal RNA.

## Introduction

Human cells contain many mitochondria and each mitochondrion several mitochondrial DNA (mtDNA) molecules. The absence of mtDNA genetic variation in a person is named homoplasmy. However, homoplasmic mutations can be found in a particular individual when his/her mtDNA is different from a reference sequence, the revised Cambridge Reference Sequence (rCRS). If these mutations do not have phenotypic effect, they can survive in the populations for long periods of time. On the contrary, if mutations have very dramatic functional effects, they will be very quickly removed from the populations. In between, there are several pathologic mtDNA mutations with moderate phenotypic effects. These are the etiologic factors for some of the most frequent mtDNA diseases, such as the Leber hereditary optic neuropathy (LHON) and the non-syndromic hearing loss (NSHL) (Fischel-Ghodsian, 2000; Carelli et al., 2003). A common feature for these homoplasmic mutations with moderate functional effect is their tissue-specificity. For example, despite being present in every mtDNA molecule of each cell, LHON and NSHL mutations mainly affect retinal ganglion and cochlear cells, respectively. This fact suggests that tissue-specific factors, including the specific set of expressed proteins, are required for the manifestation of the pathologic phenotype (Hamalainen et al., 2013). It is also frequently found that maternal relatives of patients carrying homoplasmic mutations are asymptomatic. This fact, again, suggests that other environmental and genetic factors are necessary for the phenotypic expression (Fischel-Ghodsian, 2000; Carelli et al., 2003).

The m.1494C>T mutation in the 12S rRNA gene (*MT-RNR1*) was found in several NSHL patients from a Chinese family. The oxygen consumption was lower in lymphoblastoid cell lines from homoplasmic mutant individuals (Zhao et al., 2004). Moreover, oxygen consumption and mtDNA-encoded protein synthesis was also lower in mutant transmitochondrial osteosarcoma 143B cell lines (cybrids) (Zhao et al., 2005). Therefore, the biochemical phenotype was transferred with the mtDNA. As 12S rRNA is required for the synthesis of mtDNA-encoded proteins and all these polypeptides are part of the oxidative phosphorylation (OXPHOS) system, the main oxygen consumer of the cell, these results confirmed the pathogenicity of the m.1494C>T mutation. Since then, homoplasmic m.1494C>T mutation has been reported in 14 isolated NSHL patients and 22 Chinese and 3 Spanish NSHL pedigrees with different genetic background with respect to mtDNA (Rodriguez-Ballesteros et al., 2006; Wang et al., 2006; Chen et al., 2007, 2011; Han et al., 2007; Yuan et al., 2007; Zhu et al., 2009; Wei et al., 2013a,b; Yang et al., 2013; Zhang et al., 2013). A total of 533 maternal relatives of these 26 families were studied, but 398 did not suffer from NSHL. Moreover, this mutation was also found in a normal Chinese individual in a population study to reconstruct the East Asian mtDNA phylogeny (Kong et al., 2003).

The discovery of modifying factors that alter the penetrance of mutations is not an easy task. Conventional genetic approaches such as linkage analysis might not be informative when there are many factors contributing to the disease (Carelli et al., 2003; Hudson et al., 2007; Kirkman et al., 2009; Giordano et al., 2011). We have recently found that m.1555G in the *MT-RNR1* gene, which also provokes NSHL in humans (Prezant et al., 1993), is the wild-type allele in orangutans and some other mammalian species (Pacheu-Grau et al., 2011). These mutations that have been fixed in other species are called compensated pathogenic deviations (CPD) because, at this site, a nucleotide or amino acid substitution would be pathogenic to humans and, therefore, an organism carrying a CPD must also have some other compensatory deviation with respect to humans that suppresses the deleterious effect of the CPD (Kondrashov et al., 2002).

In order to find modifying factors affecting the penetrance of the m.1494C>T transition, we looked for mammalian species with the m.1494T allele and analyzed the mitochondrial translation system and OXPHOS function.

## Materials and methods

### Bioinformatics studies

We obtained 608 mammalian *MT-RNR1* sequences pulled out from completely sequenced mtDNAs published in GenBank (http://www.ncbi.nlm.nih.gov/sites/entrez) until June 2014. We also recovered 42 other total or partial Cercopithecidae *MT-RNR1* sequences from GenBank. 70 mammalian mitochondrial ribosomal protein S12 (MRPS12) sequences were obtained from Ensembl (http://www.ensembl.org/index.html). Sequence alignments were performed using the multiple sequence alignment program ClustalW2 (http://www.ebi.ac.uk/Tools/msa/clustalw2/). Conservation indexes were estimated as the percentage of sequences harboring the human wild-type variant. To confirm the species origin of our cells, we also used Basic Local Alignment Search Tool (http://blast.ncbi.nlm.nih.gov/Blast.cgi). The molecular model for ss-rRNA/RPS12 (PDB 2WDG) was obtained with the Deep view/Swiss PDB viewer and the PyMOL Molecular Graphics System, version 1.5.0.4 Schrödinger, LLC. Human non-synonymous SNPs in MRPS12 were found in dbSNP Home Page (http://www.ncbi.nlm.nih.gov/projects/SNP/).

### Cell culture conditions

Primary fibroblasts from macaque skin explant were expanded in high-glucose (25 mM) DMEM (Gibco-Life Technologies) supplemented with glutamine, pyruvate and fetal bovine serum 20% (Gibco-Life Technologies) at 37°C in a 5% CO_2_atmosphere. For all cellular and biochemical studies, macaque cells were grown 72 h in the same medium but lacking glucose and supplemented with galactose 5 mM. Human osteosarcoma 143B cybrids were grown in high-glucose DMEM medium (Gibco-Life Technologies) supplemented with glutamine, pyruvate and fetal bovine serum 5% (Gibco- Life Technologies). When required, paromomycin (Sigma-Aldrich) 2 or 4 mg/ml was added to the cell culture.

### Kirby-Bauer disk susceptibility test

Analyses of bacterial susceptibility to paromomycin were performed following the Kirby-Bauer procedure updated by the Clinical Laboratory Standards Institute (Clinical Laboratory Standards Institute, 2006). Cell cultures of the test organism (*Escherichia coli* TG1) were grown to mid-log phase. Mueller-Hinton agar plates were inoculated with 200 μl of bacterial suspension containing 3 × 10^8^ CFU/ml. Mitochondrial preparations from immortalized macaque cells were obtained as described (Fernandez-Vizarra et al., 2010). Whatman paper discs (6 mm diameter) impregnated with 20 μl of whole cell extracts or mitochondria-enriched fractions (20 μg protein/μl in PBS) were placed on the agar surface. Inoculated plates were incubated for 14-16 h at 37°C. Sensitivity to the antibiotic was demonstrated by detecting a zone of growth inhibition.

### Human MRPS12 constructs and lentiviral transduction

cDNA was obtained by using the reverse transcription system Transcriptor First Strand cDNA Synthesis Kit (Roche), using total RNA extracted from cultured m.1555A cybrids with TRIzol reagent (Invitrogen). MRPS12 coding sequence including the 5′ UTR sequence (Ensembl Transcript ID: ENST00000407800) was PCR amplified with Fw: GTTTAAACGCCACCATGTCCTGGTCTGGCC and Rv: GTTTAAACTGTTTATTAAAACCCC primers. The obtained PCR product was cloned into the pCR2.1 TOPO-TA cloning system (Invitrogen). A sequence checked clone was used as template for site directed mutagenesis by using QuikChange® Site-Directed Mutagenesis Kit (Stratagene) and the mutagenic primers: hMRPS12-R68L Fw: CTGTGCACGTTTACCCTCAAGCCGAAGAAGCC and hMRPS12-R68L Rv: GGCTTCTTCGGCTTGAGGGTAAACGTGCACAG. Inserts with the correct sequences were subsequently cloned into the lentiviral expression vector pWPI (Tronolab) upstream of the ires-GFP coding sequence.

Lentiviral particles were generated in HEK 293T packaging cells, and human cells were transduced with the formers as described (Bayona-Bafaluy et al., 2011). Twenty-four hours after transduction, GFP fluorescence was monitored by fluorescence microscopy (Leica). GFP levels were also determined by Western blot.

### Molecular-genetics and biochemical analysis

The sequencing of macaque 12S rRNA was done as previously described (Pacheu-Grau et al., 2011). To locate mutations, the human rCRS (GenBank NC_012920) was used. The sequencing of Cercopithecidae MRPS12 was also performed as already explained (Pacheu-Grau et al., 2012), but using specific primers for these species Fw: CACTAAGATCTGTTCTCTGCC and Rv: ACTCCACAAGGGTTCACATC. To locate mutations, the *Macaca mulatta* MRPS12 cDNA (Ensembl Transcript Id: ENSMMUT00000038520) was used. Overexpressed variants of this gene were sequenced from cDNA with the same primers used for cloning. The MRPS12 mRNA levels were determined by quantitative PCR assays that were carried out in a LightCycler 2.0 system (Roche), using FastStart DNA MasterPLUS SYBR Green I (Roche) and primers qMRPS12-36 Fw: AGGCAGCCACTCATGGATT, qMRPS12-36 Rv: GGCTTAATAGTGGTCCTGATGG.

For the determination of respiratory complex IV (CIV) activity and quantity (Mitoprofile® Human Complex IV Activity and Quantity, Mitosciences, Invitrogen) CIV is immunocaptured within the wells and activity is determined colorimetrical by the oxidation of reduced cytochrome c as an absorbance decrease at 550 nm. Subsequently, in the same wells the quantity of enzyme is measured by adding a CIV specific antibody conjugated with alkaline phosphatase. The phosphatase changes a substrate from colorless to yellow at 405 nm. Other molecular analyses, such as p.MT-CO1 levels, Western blots, or mitochondrial protein synthesis, were performed according protocols previously described (Gomez-Duran et al., 2010; Pacheu-Grau et al., 2011, 2013). For Western blots, primary antibodies were against p.MT-CO1 (1:1000, 459600, Life Technologies), Actin (1:2000, A2066, Sigma), TOM20 (1:200, sc-11415, Santa Cruz Biotechnology), MRPS12 (1:2000, AV46301, Sigma) and p.MT-CO1 and SDHA together (MitoBiogenesis™ In-Cell ELISA Kit (IR), ABCAM ab110216).

The cell viability was measured using the Neutral Red kit (Sigma-Aldrich) and conditions previously published (Repetto et al., 2008). Briefly, cells were either treated with paromomycin 2 or 4 mg/ml or left untreated for 72 h. Then, the cells were incubated for 2 h with neutral red dye (3.3 g/l), washed with PBS and treated with elution medium followed by gentle shaking for 10 min. Aliquots of the resulting solutions were transferred to 96-well plates, and the absorbance at 540 nm was recorded using a NovoStar MBG Labtech microplate instrument.

### Statistical analyses

The statistical package StatView 6.0 was used to perform all the statistics. Data for mean, standard deviation and number of analysis or replicates are presented. The unpaired two-tailed *t*-test was used to compare parameters. *P*-values lower than 0.05 were considered statistically significant.

## Results

### The human pathological allele m.1494T is fixed in Cercopithecidae

To search for the presence of m.1494T in mammalians, we aligned the *MT-RNR1* gene of 608 mammalian species obtained from complete mtDNA sequences of the NCBI RefSeq database. This NSHL mutation was the wild-type allele in 64 of them and, in particular, in 47 of 48 primates of the Cercopithecidae family (Supplementary Material: Note 1). To corroborate that this pathological mutation is the wild-type allele in primates from this family, we extended our analysis to include 42 other *MT-RNR1* sequences from different species of the Cercopithecidae family obtained from non-complete mtDNA sequences. These 90 species include members of the four tribes (Colobini -3- and Presbytini -21-, Cercopithecini -32-, and Papionini -34-) from the two subfamilies (Colobinae and Cercopithecinae) of the Cercopithecidae family (Perelman et al., 2011). All these species, except *Papio ursinus*, contain thymine at m.1494 position (Supplementary Material: Note 2). Therefore, we confirm that m.1494T is the wild-type allele in this family. However, the auditory function in these monkeys approaches that of humans, including middle ear, cochlear and neural function (Fowler et al., 2010; Joris et al., 2011; Dylla et al., 2013).

### The m.1494C nucleotide is important for the 12S rRNA function

The m.1494C>T pathological mutation was probably fixed in Cercopithecidae because it produces a post-reproductive phenotype and negative or purifying selection could not remove it from the population. However, several deaf individuals of the previously described human pedigrees lost their hearing capacity in reproductive years (Zhao et al., 2004; Rodriguez-Ballesteros et al., 2006; Wang et al., 2006; Chen et al., 2007; Han et al., 2007; Yuan et al., 2007; Zhu et al., 2009; Wei et al., 2013b).

MtDNA is inherited exclusively by maternal lineage and it remains possible that deafness due to a pathological mutation is a male-specific phenotype. In this scenario, negative selection acting on males would not affect m.1494T population frequency in the next generation (Frank and Hurst, 1996). However, 76 NSHL individuals from these pedigrees were female (Zhao et al., 2004; Rodriguez-Ballesteros et al., 2006; Wang et al., 2006; Chen et al., 2007; Han et al., 2007; Yuan et al., 2007; Zhu et al., 2009; Wei et al., 2013b).

These observations suggest that maternally inherited NSHL phenotype might be under selective pressure. In fact, the m.1494C position is conserved in 544 (89.5%) of 608 mammalian species examined. This conservation index (CI) is higher than the mean CI (77.6%) of the *MT-RNR1* gene, considering the CI of every position. Therefore, m.1494C nucleotide is functionally important.

### Cercopithecidae cells are resistant to aminoglycosides

It has been shown that aminoglycosides trigger NSHL in humans harboring the m.1494C>T mutation. Thus, 50 out of 135 NSHL individuals from these pedigrees became deaf after a confirmed aminoglycosides exposure (Zhao et al., 2004; Rodriguez-Ballesteros et al., 2006; Wang et al., 2006; Chen et al., 2007; Han et al., 2007; Yuan et al., 2007; Zhu et al., 2009; Wei et al., 2013b). In the bacterial rRNA the equivalent position to human m.1494 forms a Watson-Crick base pair (W-C bp) with the nucleotide in equivalent position to human m.1555, which are both important for aminoglycoside binding. We have previously shown that aminoglycosides negatively affect OXPHOS function of orangutan cells, which harbor the m.1555G allele and the W-C bp, by decreasing the synthesis of mtDNA-encoded polypeptides and the CIV amount and activity. These drugs also decrease the growth rate of orangutan cells (Pacheu-Grau et al., 2011). Human wild-type 12S rRNA lacks this W-C bp, but m.1494C>T mutation rebuilds it. Thus, mutant human 12S rRNA is more similar to the bacterial rRNA and favors aminoglycoside binding, which may produce toxic side-effects. Therefore, the m.1494C>T mutation was likely fixed as the wild-type allele in Cercopithecidae because they are not naturally exposed to aminoglycosides.

**Figure 1:**
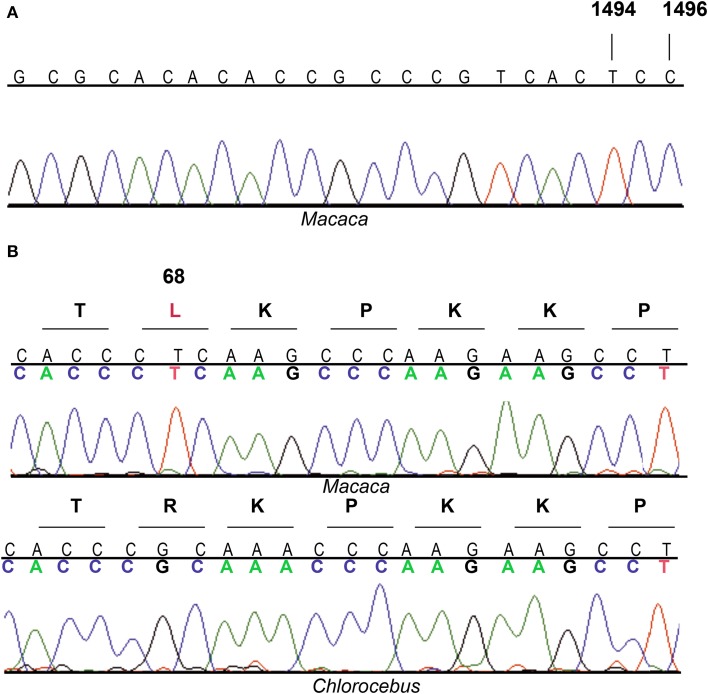
**Electropherograms of *MT-RNR1* and *MRPS12* partial sequences**. **(A)** *MT-RNR1* from *Macaca* sp. **(B)** *MRPS12* from *Macaca* sp. and *Chlorocebus aethiops*.

To analyze the effect of aminoglycosides on OXPHOS function of Cercopithecidae species, we first asked for primary fibroblasts derived from skin explant from *Macaca nemestrina* (Papionini tribe) and confirmed the origin of these cells and the existence of the mutation at m.1494 position. The sequencing of a *MT-RNR1* segment showed the presence of m.1494T nucleotide (Figure 1A). In mammals, apart from Cercopithecidae family and mutant humans, the m.1494T nucleotide has only been described in 17 species from four different families (Notoryctidae, Phascolarctidae, Elephantidae and Spalacidae) (Supplementary Material: Note 1). Another transition, m.1496T>C, is found in all species from the Cercopithecinae subfamily and the Colobinae *Procolobus verus*, but it is not found in any other mammalians with m.1494C>T mutation. The Cercopithecidae origin of our sample is supported by the m.1496T>C transition (Figure 1A). Moreover, the match percentage between this *MT-RNR1* segment (276 nucleotides, human 1302–1575 nucleotide positions) and the one from *Macaca nemestrina* was 97%. However, this match percentage reached 99% with that segment from *Macaca silenus*, the phylogenetically closest species (Morales and Melnick, 1998; Deinard and Smith, 2001; Perelman et al., 2011).

It has been reported that human immortalized lymphoblastoid cell lines and osteosarcoma 143B cybrids harboring the m.1494C>T mutation showed a decreased growth rate when cultured with 2 mg/ml of the aminoglycoside paromomycin (Zhao et al., 2004, 2005). Human immortalized lymphoblastoid cell lines and osteosarcoma 143B cybrids harboring the m.1555A>G mutation, as well as orangutan primary fibroblasts from skin explant containing m.1555G as the wild-type allele, also showed a decreased growth rate and synthesis of mtDNA-encoded proteins when cultured with 2 mg/ml of paromomycin (Guan et al., 2000; Giordano et al., 2002; Pacheu-Grau et al., 2011). However, human osteosarcoma 143B cybrids and bonobo primary fibroblasts from skin explant without any of these mutations were resistant to 4 mg/ml of paromomycin (Pacheu-Grau et al., 2011). Therefore, we used 2 or 4 mg/ml of paromomycin to analyze the effect of aminoglycosides on macaque primary fibroblasts from skin explant.

**Figure 2:**
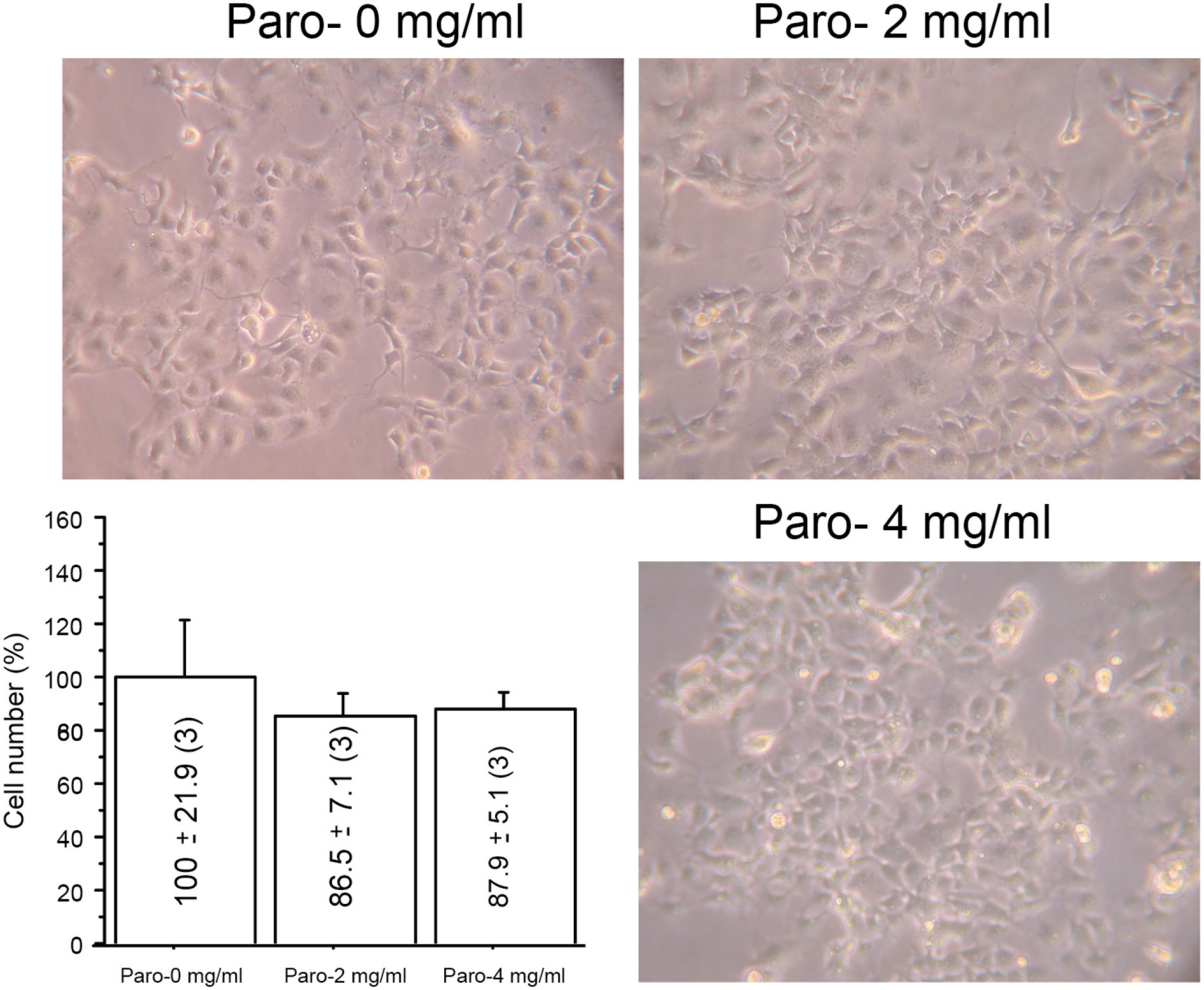
**Paromomycin effect on macaque cells**. Representative images of macaque fibroblasts growing with different amount of paromomycin (paro) and growth quantification. The number of cells after 3 days growing without aminoglycosides has been considered 100%.

When oxidative metabolism is impaired, cells cultured in galactose medium cannot synthesize the bulk of their ATP requirements by glycolysis and the cell growth is inhibited (Robinson et al., 1992). For this reason, we grew macaque cells without or with paromomycin (2 or 4 mg/ml) in galactose medium, but we did not observe any differences in the growth rate after 3 days (Figure 2).

**Figure 3:**
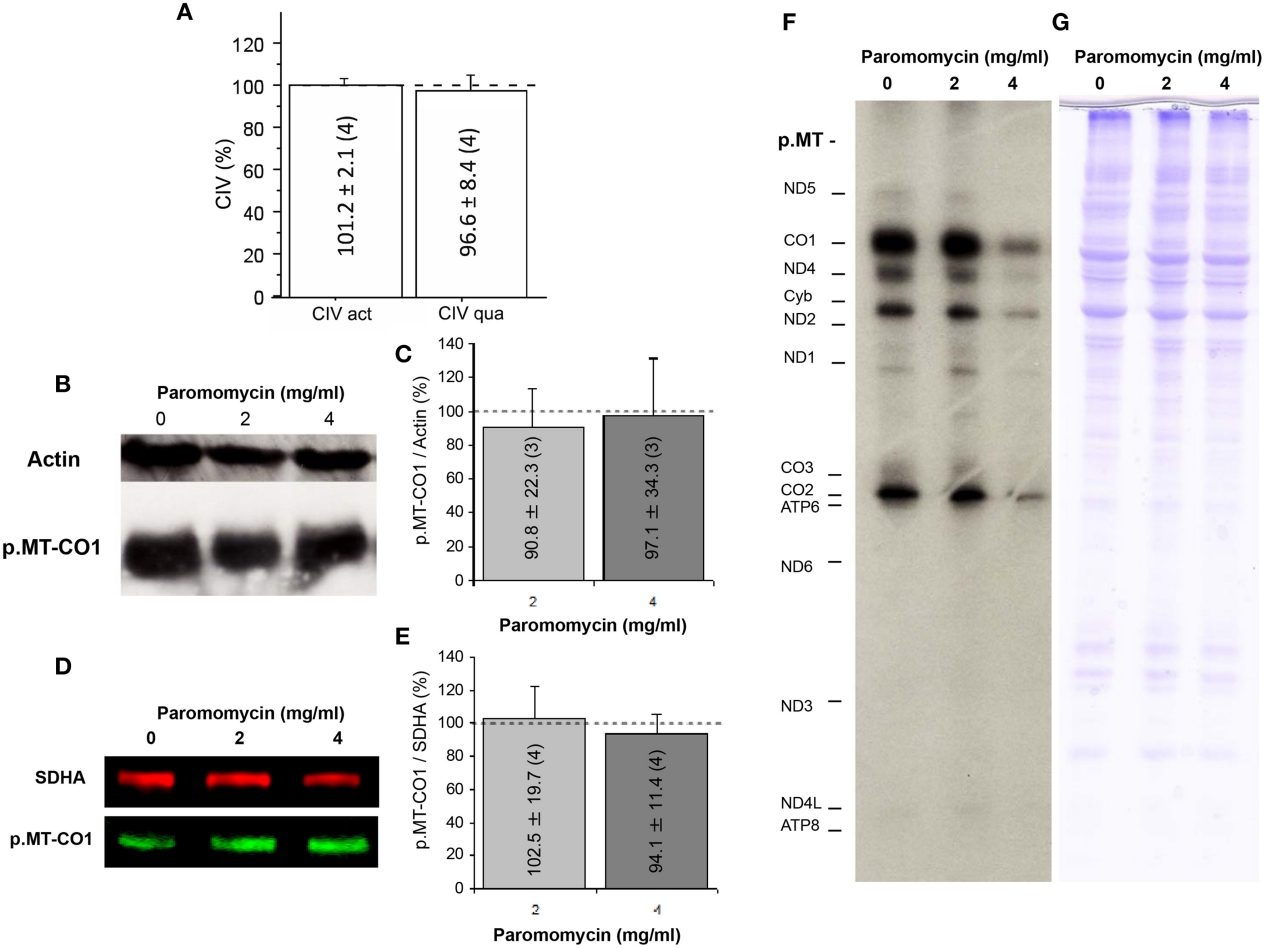
**Paromomycin effect on OXPHOS complexes**. **(A)** CIV specific activity and quantity after treatment with paromomycin 2 mg/ml. Dots line represents values in the absence of the antibiotic. **(B)** p.MT-CO1 and actin levels from macaque cells treated with paromomycin 0, 2, or 4 mg/ml. **(C)** Semiquantitative determination, by gel scanning, of p.MT-CO1/actin ratios from macaque cells treated with paromomycin 0 (dot line), 2 or 4 mg/ml. **(D)** Representative image of p.MT-CO1 and SDHA levels from macaque cells treated with paromomycin 0, 2 or 4 mg/ml. **(E)** Quantitative determination, by ELISA, of p.MT-CO1/SDHA ratios from macaque cells treated with paromomycin 0, 2 or 4 mg/ml. **(F**,**G)** Representative images of mitochondrial translation products, and loading controls, from macaque cells treated with paromomycin 0, 2, or 4 mg/ml.

To elucidate the effect of paromomycin on OXPHOS function, we determined CIV specific activity and levels, since catalytic subunits of this complex are translated in mitochondrial ribosomes and are also required for CIV assembly. We did not observe differences in these parameters when cells were grown with or without the antibiotic (Figure 3A). The p.MT-CO1 polypeptide, an mtDNA-encoded CIV subunit, was expressed at similar levels in macaque cells grown without or with paromomycin (2 or 4 mg/ml) (Figures 3B–E). Finally, the assays of mtDNA-encoded protein synthesis did not differ between cells grown without or with 2 mg/ml paromomycin, although the levels of mtDNA-encoded polypeptides were lower when cells were grown with 4 mg/ml of paromomycin (Figures 3F,G).

**Figure 4:**
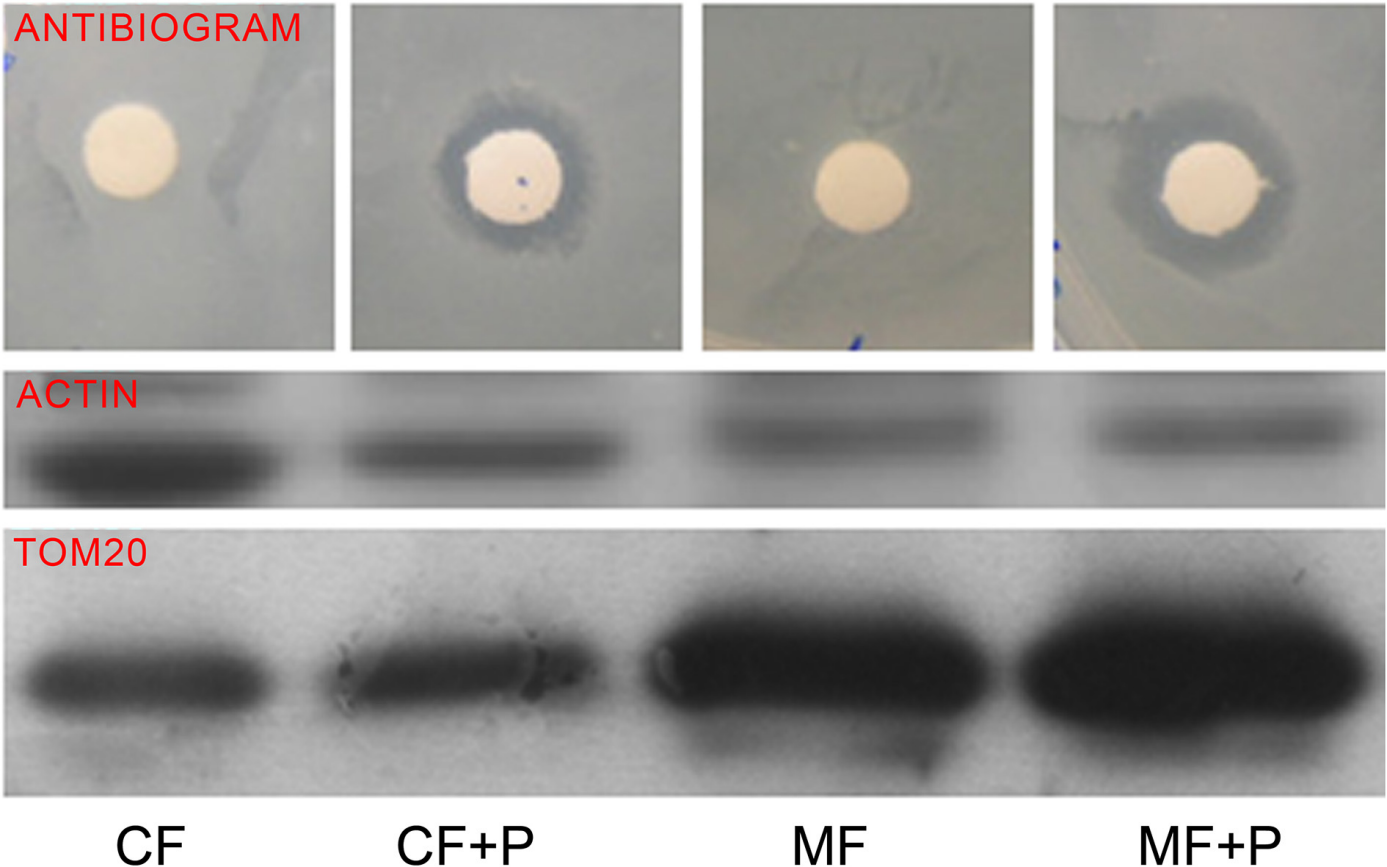
**Mitochondrial fraction antibiogram**. TOM20 is a mitochondrial protein. CF, CF+P, MF, and MF+P code for cell fraction, cell fraction from cells treated with paromomycin 2 mg/ml, mitochondrial fraction and mitochondrial fraction from cells treated with paromomycin 2 mg/ml, respectively.

To confirm that the antibiotic was incorporated into mitochondria, we performed an antibiogram of a mitochondria-enriched fraction. The mitochondrial fraction showed a higher inhibition of the bacterial growth than the cell homogenate (Figure 4). This, along with the effect on mtDNA-encoded protein synthesis by high concentrations of paromomycin, supports the presence of this antibiotic in the mitochondrial matrix.

These results indicate that paromomycin does not have important effects on OXPHOS function of macaque cells and suggest that Cercopithecidae cells are more resistant to paromomycin. Therefore, m.1494C>T mutation should be compensated in this species. Compensation would explain why auditory function in macaques (*Macaca nemestrina*) and vervet monkeys (*Chlorocebus pygerythrus*) was essentially unaffected by dihydrosptreptomycin (Stebbins et al., 1987), although the patas monkey (*Erythrocebus patas*), another member of Cercopithecidae, showed an important susceptibility to the ototoxic action of dihydrostreptomycin 20 mg/kg for 90 days (Hawkins et al., 1977). It is remarkable that hearing damage is nearly universal when high drug levels are present for prolonged periods (Fischel-Ghodsian, 2003). The abundance of positively charged amino groups predestines aminoglycosides to bind negatively charged molecules, such as other non-ribosomal RNAs or phospholipids (Hermann, 2007; Hainrichson et al., 2008). Therefore, the ototoxic effect in patas monkey could be due to different mechanisms at high and prolonged aminoglycoside doses.

### A MRPS12:R68L substitution could compensate the pathological mutation m.1494C>T in macaque

**Figure 5:**
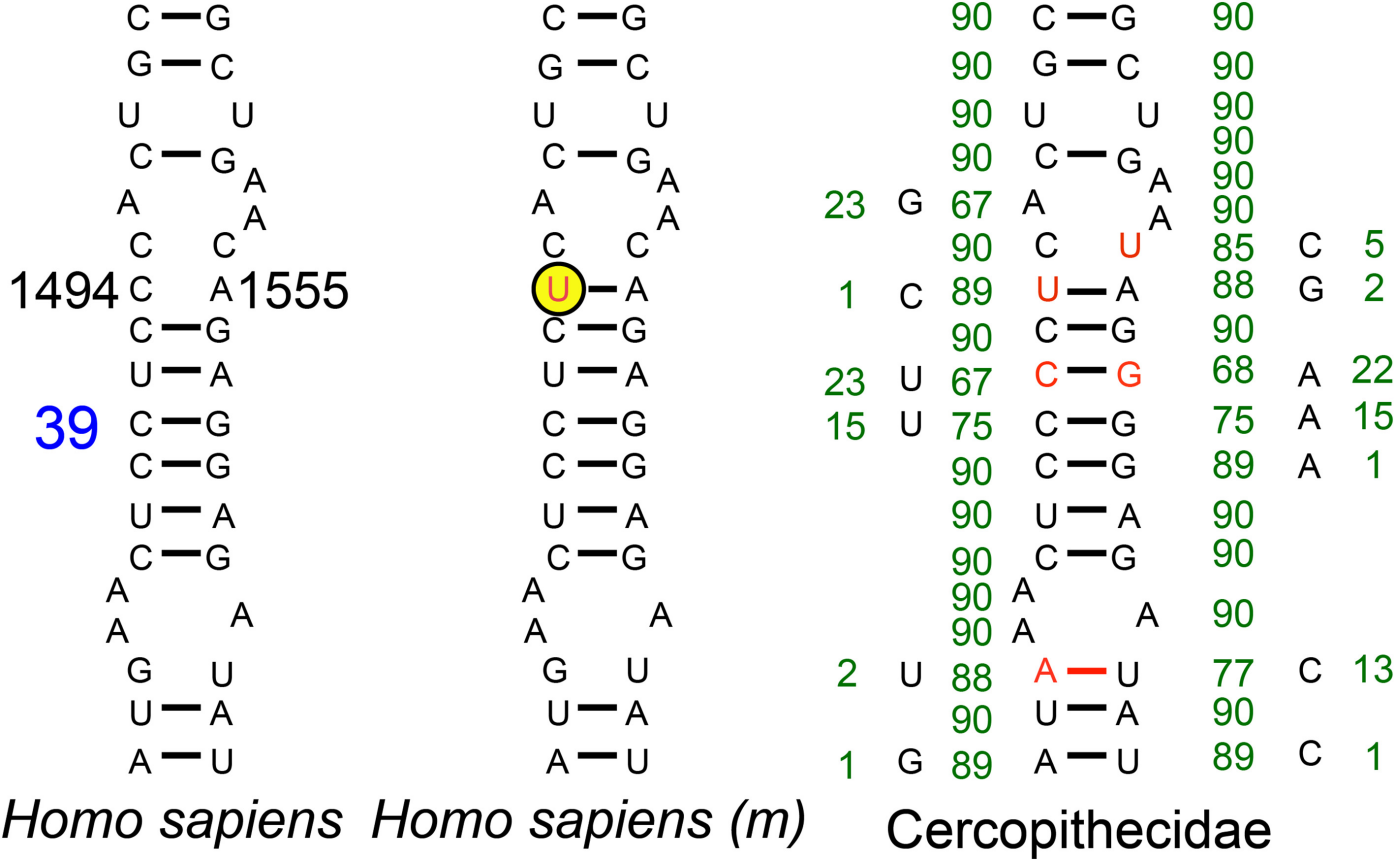
**Helix 39 of the 12S rRNA decoding site from Human and Cercopithecidae**. Blue number denotes helix numbering (Springer and Douzery, 1996). The m.1494C>T transition, in the yellow-encircled nucleotide position, is a human pathologic mutation (m). Red nucleotides denote differences vs. the human sequence. Green numbers indicate the absolute frequency of that nucleotide in 90 Cercopithecidae species. Several mutations have been compensated in this helix 39.

Compensatory mutations have been proposed as probable explanations for fixation of disease mutations in mtDNA-encoded protein and tRNA genes (Kern and Kondrashov, 2004; Azevedo et al., 2009). Compensatory mutations are also frequently found in mtDNA-encoded rRNAs (Springer et al., 1995). As compensatory mutations usually appear in the same gene product as the CPD (Kondrashov et al., 2002), we analyzed the 12S rRNA decoding site in Cercopithecidae. However, despite the existence of compensatory mutations at helix 39 of the decoding site from the Cercopithecidae rRNA, the m.1494C>T pathologic mutation has not been compensated by an m.1555A>G transition (Figure 5).

**Figure 6:**
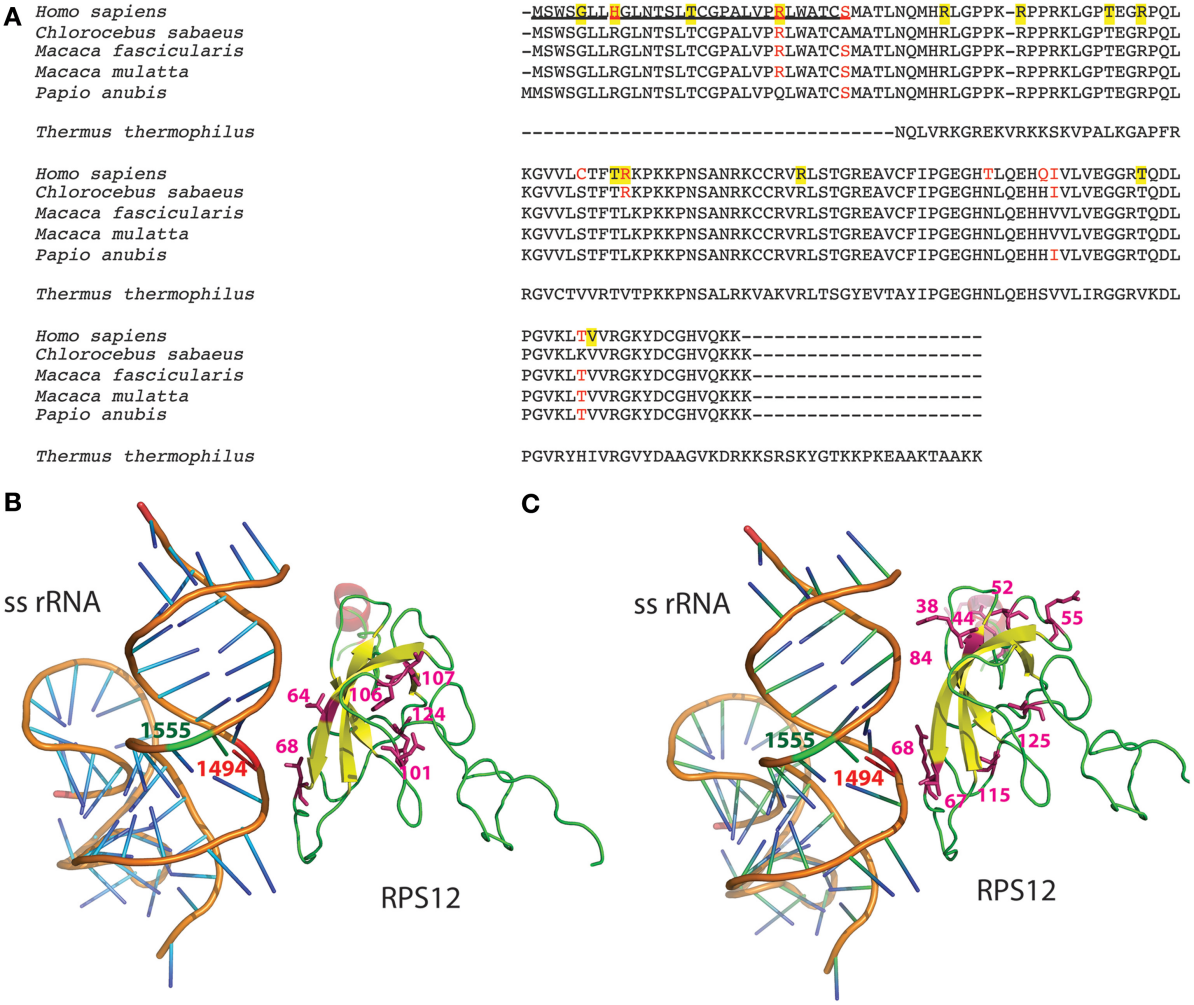
**MRPS12′s amino acid variation**. **(A)** Protein alignment. MRPS12 sequences from 4 Cercopithecidae species, *Homo sapiens* and *Thermus thermophilus*. Red amino acids denote differences between human and Cercopithecidae species. Yellow background indicates human polymorphic positions. Underlined sequence is the mitochondrial targeting sequence. **(B,C)** Molecular view of the interaction between the small subunit rRNA (ss rRNA) and the ribosomal protein S12 in the *Thermus thermophilus* ribosome. The mitochondrial 12S rRNA nucleotides (1494 and 1555, red and green, respectively) and the MRPS12 amino acid variation between human and macaque sequences (64, 68, 101, 106, 107, and 124) or the human MRPS12 polymorphic amino acids (38, 44, 52, 55, 67, 68, 84, 115, and 125) have been located in this bacterial structure.

Bacterial homolog of MRPS12, 30S ribosomal protein S12 (Rps12), is a critical component of the ribosomal decoding center (Vila-Sanjurjo et al., 2007; Demirci et al., 2013). Compensatory interactions between this ribosomal protein and bacterial rRNA variants have already been reported (O'Connor et al., 1991). We compared the human MRPS12 sequence with that from four Cercopithecidae species and observed six variable amino acids in the mature protein (Figure 6A). To confirm their functional significance, we estimated their CIs using 70 mammalian species (Supplementary Material: Note 3). The C64, R68, T101, Q106, I107, and T124 CIs were 7.1, 90.0, 24.3, 10.0, 24.3, and 55.7%, respectively. R68, with the highest CI, is present in 63 out of 70 mammalian species, including *Chlorocebus sabaeus* (*Cercopithecus sabeus*), a member of the Cercopithecini tribe. However, 3 species from the Papionini tribe contain L68 (Figure 6A). To confirm the presence of L68 in the macaque MRPS12 protein and its absence in members of the Cercopithecini tribe, we sequenced this gene in the macaque primary fibroblasts and the *Chlorocebus aethiops* COS-1 cell line (GenBank: KM885248 and KM885249) (Figure 1B). Between our macaque sequence and the one from *Macaca mulatta* or *Macaca fascicularis* we found one amino acid difference (V107I). We found no amino acid differences between the *Chlorocebus aethiops* sequence and the one from *Chlorocebus sabaeus* (Figure 6A). Thus, the MRPS12:L68 was found in four species of the Papionini tribe but it was not present in two of the Cercopithecini tribe.

To check potential interactions between these MRPS12 amino acids and the 12S rRNA m.1494 nucleotide, we located them in the crystal structure of a bacterial (*Thermus thermophilus*) ribosome (Figure 6A). The position equivalent to amino acid 68 is located less than 6 Å from m.1494 nucleotide whereas the other polymorphic positions are far away from m.1494 (Figure 6B). Moreover, some mutations in *Escherichia coli* Rps12, equivalent to positions 64, 68, 106, and 124 in mammals, provide a restrictive phenotype that confers resistance to the error-inducing aminoglycosides (Maisnier-Patin et al., 2007; Agarwal et al., 2011). Therefore, these potential aminoglycoside-resistance mutations in macaque MRPS12 could possibly compensate the aminoglycoside-susceptibility mutation in 12S rRNA.

### Genetic MRPS12 variation as a modifier factor to explain the incomplete penetrance of m.1494C>T or m.1555A>G transitions in humans

The results above suggested that variation in MRPS12 could be a phenotype-modifying factor for mtDNA-inherited NSHL mutations. In humans, 15 non-synonymous MRPS12 variants have been described (4 of them in the mitochondrial targeting sequence: rs143032757, G5S; rs33988199, H8R; rs199548036n T15I and rs150096976, R23W and 11 in the mature protein: rs375680427 and rs201447868, R38C and R38H; rs147007310 and rs375471558, R44G and R44Q; rs368555992, T52M, rs374146379, R55Q; rs111688400, T67S; rs147184696, R68C; rs140018981, R84W; rs202014859, T115A; and rs143918075, V125I). To assess their functional importance, we estimated the CIs for R38, R44, T52, R55, T67, R68, R84, T115, and V125 in 70 mammalian species (Supplementary Material: Note 3). Their CIs were 92.9, 41.4, 92.9, 95.7, 28.6, 90.0, 94.3, 97.1, and 87.1%, respectively. The mean CI of these variant positions (80%) is significantly higher than that of the Cercopithecidae variants (35.2%). Then, to check potential interactions between these MRPS12 amino acids and the 12S rRNA m.1494 nucleotide, we located them in the crystal structure of a bacterial (*Thermus thermophilus*) ribosome (Figure 6C). As previously mentioned, the bacterial protein position equivalent to 68-amino acid is located less than 6 Å from m.1494 nucleotide but the other human polymorphic positions reside far away from m.1494. Mutations in the *Escherichia coli* Rps12 equivalent to positions 52 and 68 in mammals confer resistance to error-inducing aminoglycosides (Agarwal et al., 2011). Therefore, several MRPS12 positions are possible modifiers for *MT-RNR1* mutations in humans.

### The MRPS12:R68L substitution modifies the phenotype of the pathological mutation m.1555A>G in cultured cells

Ideally, to confirm a modifying role for an amino acid substitution in the MRPS12 in mt-rRNA inherited NSHL, homoplasmic mutant maternal relatives suffering or not aminoglycosides triggered deafness should be genotyped. However, in none of the published pedigrees it was reported whether hearing individuals were treated with aminoglycosides (Zhao et al., 2004; Rodriguez-Ballesteros et al., 2006; Wang et al., 2006; Chen et al., 2007; Han et al., 2007; Yuan et al., 2007; Zhu et al., 2009; Wei et al., 2013b). In a previous work, we sequenced the MRPS12 protein in 21 Ménière disease patients who suffered a chemical labyrinthectomy by using gentamycin. Ten became deaf and eleven retained their hearing ability. However, they did not have the mt-rRNA pathogenic mutation and we did not discover amino acid substitutions that could explain the phenotype (Pacheu-Grau et al., 2012).

**Figure 7:**
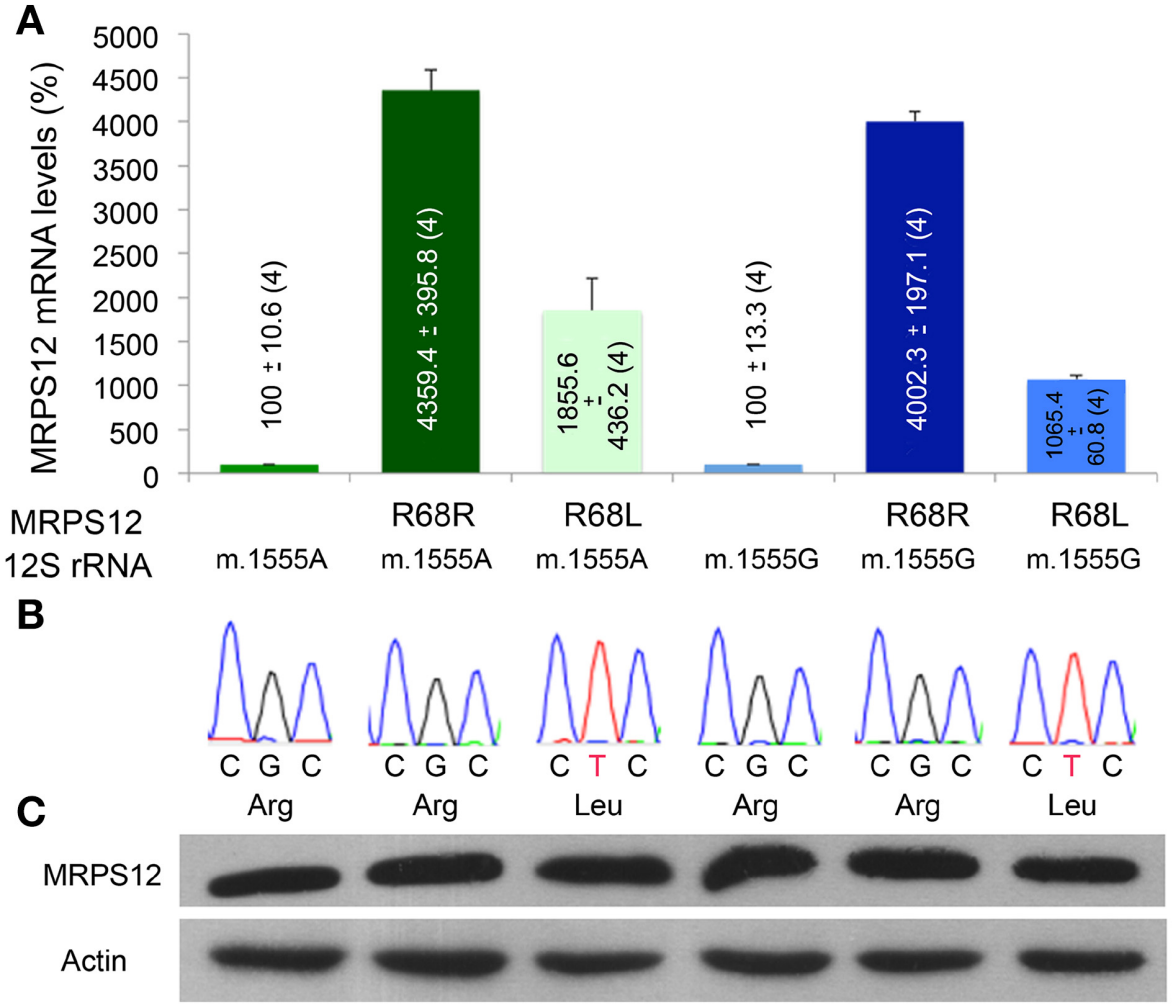
**MRPS12 expression**. **(A)** MRPS12 mRNA levels. The levels of non-transfected cells are considered 100%. **(B)** MRPS12 codon 68. Leu and Arg code for leucine and arginine, respectively. **(C)** Relative amount of MRPS12 and Actin proteins.

**Figure 8:**
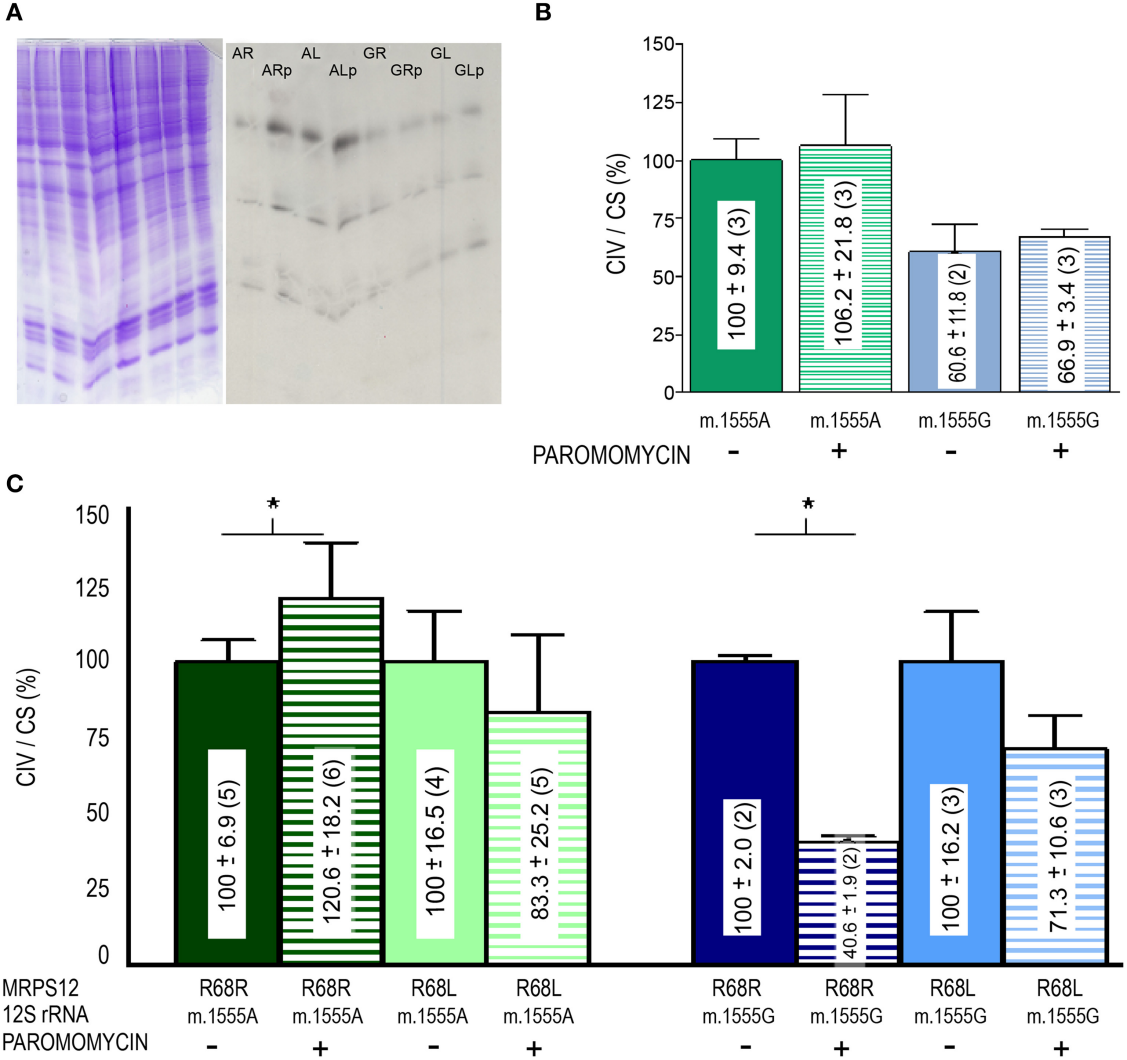
**Paromomycin effect on OXPHOS proteins of MRPS12 transfected cells**. **(A)** Mitochondrial translation products (right panel) and loading controls (left panel). AR, AL, GR, GL, ARp, ALp, GRp, and GLp code for 12S rRNA m.1555A-MRPS12^R68R^, 12S rRNA m.1555A-MRPS12^R68L^, 12S rRNA m.1555G-MRPS12^R68R^, 12S rRNA m.1555G-MRPS12^R68L^ cells grown without or with paromomycin (p), respectively. **(B)** CIV/CS ratio of wild-type (m.1555A 12S rRNA) and mutant (m.1555G 12S rRNA) osteosarcoma 143B cybrids exposed or not to paromomycin. **(C)** CIV/CS ratio of wild-type (m.1555A 12S rRNA) and mutant (m.1555G 12S rRNA) osteosarcoma 143B cybrids that overexpress the R68 or L68 MRPS12 variants exposed or not to paromomycin. Asterisks denote *P* ≤ 0.0414.

To confirm the potential modifying role of this MRPS12:R68L variant, human cybrid cell lines harboring either the m.1555A or the m.1555G alleles were transfected with the human wild-type MRPS12 cDNA or its mutated version harboring the macaque L68 substitution. The MRPS12 mRNA levels were at least 10 times higher than those of the non-transfected cells (Figure 7A). Sequence analysis of RT-PCR products derived of these mRNAs showed the presence of only one type of mRNA. Sanger sequencing is not sensitive enough to detect the low amounts of cDNA raising from the endogenous allele, which is masked by the huge amount of cDNA raising from the transfected transgene (Figure 7B). The levels of the MRPS12 polypeptide were similar in all the transfected cell lines (Figure 7C). These cell lines were grown in the presence or absence of paromomycin and mitochondrial synthesis of proteins was evaluated. However, the assay is semiquantitative and did not provide a clear result (Figure 8A). Then, we determined CIV activity normalized by citrate synthase (CS) activity. This ratio was significantly higher (*P* = 0.027) in non-transfected wild-type than mutant cells but the aminoglycoside did not have a significant effect on any of these activities (Figure 8B), confirming a similar result by others (Giordano et al., 2002). On the other hand, the ratio was significantly higher in paromomycin-treated cells that harbor the m.1555A 12S rRNA allele and overexpress the R68 MRPS12 variant (12S rRNA m.1555A-MRPS12^R68R^) than in the same cells without paromomycin (Figure 8C). However, this activity was significantly lower in paromomycin-treated cells that harbor the m.1555G 12S rRNA allele and overexpress the R68 MRPS12 variant (12S rRNA m.1555G-MRPS12^R68R^) than in the same untreated cells (Figure 8C). Taken together, the results above showed that the R68 or L68 MRPS12 overexpression in wild-type (m.1555A 12S rRNA) or mutant (m.1555G 12S rRNA) cells, respectively, increases the resistance against paromomycin. There is, apparently, a combination of 12S rRNA and MRPS12 positions that protect against aminoglycosides. Thus, the W-C bp 1494–1555 along with L68 MRPS12 or the lack of W-C bp in these positions along with R68 MRPS12 increases the resistance to paromomycin.

## Discussion

We have shown that an amino acid substitution in the MRPS12 position 68 can compensate functional effects of 12S rRNA pathologic mutations m.1494C>T and m.1555A>G.

**Figure 9:**
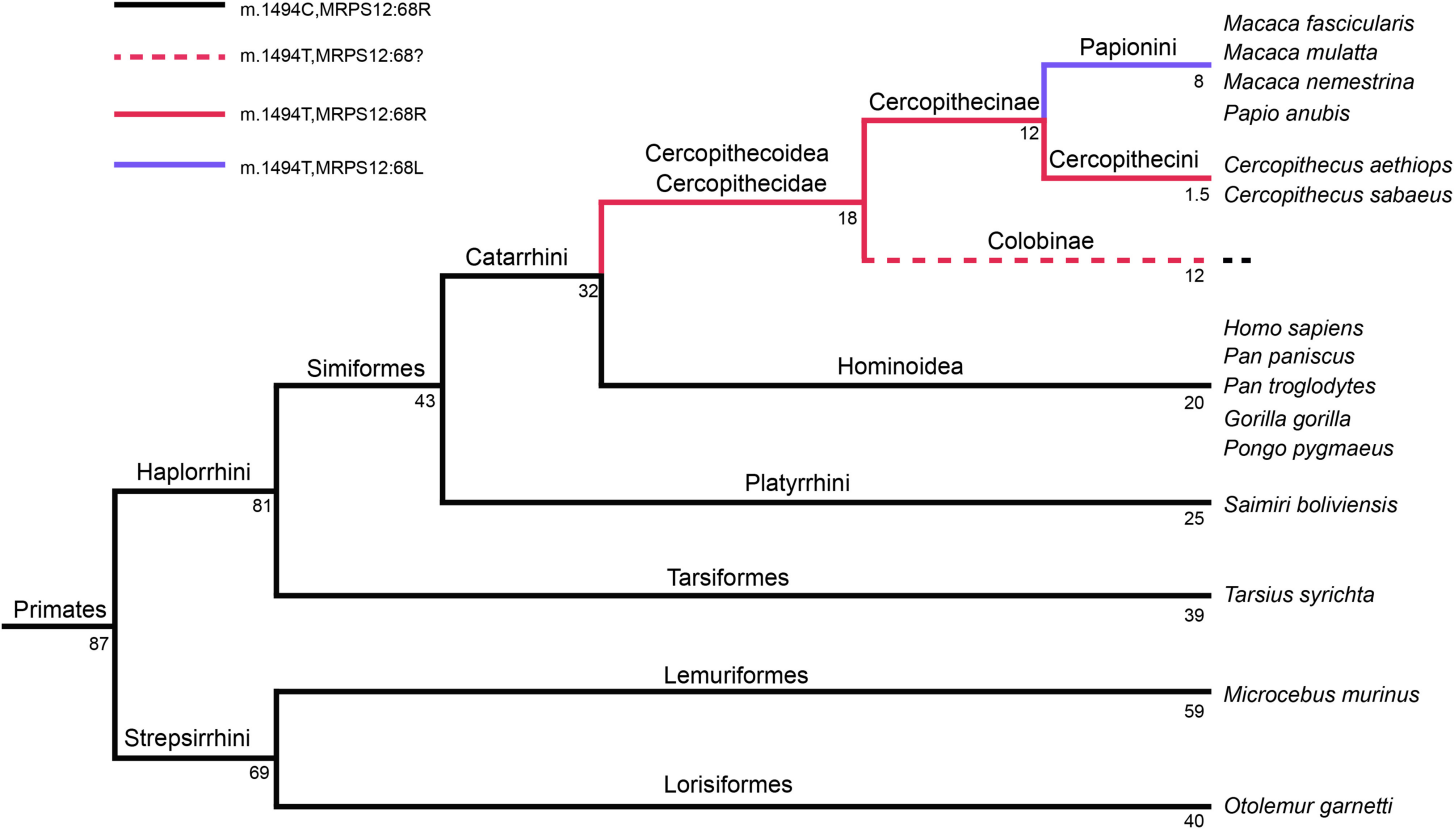
**Primates' phylogeny**. Numbers indicate million of years. This tree is based in Figure 2 from Perelman et al. (2011).

Several scenarios have been proposed to explain the co-occurrence of CPDs and compensatory mutations. In the first one, it has been suggested that a compensatory mutation could be phenotypically neutral and stable, thus fixing itself quickly in the population. On the other hand, a pathogenic mutation would be unstable and become fixed only if it would occur after the compensation mutation (Depristo et al., 2005). However, the possible MRPS12:R68L compensatory mutation appears to be an aminoglycoside-resistance mutation. Aminoglycosides produce misreading of the genetic code. In bacteria, this mutation would increase the accuracy of translation but the improved fidelity in such mutants is offset by their typically slower growth and decreased rates of protein synthesis (Agarwal et al., 2011). Resistance to antibiotics is usually associated with a fitness cost (Maisnier-Patin and Andersson, 2004). Therefore, this mutation may not be phenotypically neutral, would be target of negative selection and be removed before the appearance of the m.1494 mutation. In the second scenario, both CPD and compensatory mutations are individually deleterious, but together have a neutral effect (Depristo et al., 2005). Therefore, they should be simultaneously present in the population. Most of mtDNA pathologic mutations are recessive and require a certain percentage of heteroplasmy (mix of wild-type and mutant mtDNA genomes) to produce a phenotype. Moreover, nuclear DNA (nDNA) mutations can be found in heterozygosity. These two conditions, heteroplasmy and heterozygosity, may allow the fixation of both mutations at the same time. However, the presence of m.1494T in all Cercopithecidae but the absence of MRPS12:L68 in monkeys from the Cercopithecini tribe suggests that the mitochondrial pathologic transition was older than the nuclear compensation (Figure 9). Because Hominoidea diverged from Cercopithecoidea 32 million years ago and divergence time for Cercopithecoidea species is 18 million years (Perelman et al., 2011), the range of age for the appearance of this m.1494C>T mutation is 32–18 million years ago. However, the MRPS12:R68L compensatory mutation had to be fixed between 12 (it is not present in Cercopithecini) and 8 million years ago (it is found in Papionini tribe that diverged 8 million years ago) (Perelman et al., 2011). Thus, at least 6 million years passed between the fixation of these mt-rRNA and MRPS12 mutations. Up to now, there are not any other published Cercopithecini MRPS12 sequences out of those for two *Chlorocebus* (*Cercopithecus*) species, and we could not genotype any member of the Colobinae subfamily. If Colobinae species harbor a MRPS12 L68 amino acid, it would be possible that both 12S rRNA m.1494C>T and MRPS12 R68L mutations appeared at the same time, but the MRPS12 position reverted in the *Chlorocebus* species.

Considering that the previous scenario may be wrong, how can we explain that the compensatory mutation occurred after the fixation of the deleterious mutation? A potential explanation, functional epistasis, has been suggested for a homoplasmic pathologic mutation that affects another important component of mitochondrial translation system, the tRNA^Ile^. This mutation can induce a compensatory response by increasing mitochondrial biogenesis. Those individuals that are not able to have this compensatory response developed a mitochondrial disease but some women harboring this mutation would be able to increase mitochondrial biogenesis, survive, reproduce, and transmit the mutation to their descendants. The same options would be open for individuals of the next generation. In this way, the probability to fix another mutation that structurally compensated the pathologic one would increase (Moreno-Loshuertos et al., 2011).

Independently of the mechanism to fix a compensatory mutation of a CPD, an analysis of MRPs' evolution suggests that genetic compensation is probably very common. Despite mtDNA is evolving much faster than nDNA, MRPs are evolving at a rate comparable to that of mt-rRNAs. MRPs also evolve at a higher rate than cytosolic ribosomal proteins although both cytosolic and mitochondrial sets of ribosomal proteins are encoded by nDNA genes. Comparison of the ribosomal protein and rRNA rates of evolution suggests that the changes are being fixed at comparable rates, despite the different mutational rates for the RNA and protein and for the two genomes (Matthews et al., 1978; Pietromonaco et al., 1986). Similar to the high evolution rates of nDNA-encoded MRPs to accommodate the mtDNA-encoded rRNAs evolution rates (Pietromonaco et al., 1986), nDNA-encoded mitochondrial aminoacyl-tRNA synthetases have also higher evolution rates than nDNA-encoded cytosolic aminoacyl-tRNA synthetases to accommodate the mtDNA-encoded tRNAs evolution rates (Brindefalk et al., 2007; Frenkel-Morgenstern et al., 2009). Moreover, nDNA-encoded OXPHOS complex subunits evolve faster than other nDNA-encoded proteins to accommodate the evolution of mtDNA-encoded OXPHOS complex subunits (Osada and Akashi, 2012). Therefore, genetic variants of nDNA frequently compensate pathologic mtDNA mutations. In this sense, m.1494C>T and m.1555A>G are pathologic human mutations with incomplete penetrance and one potential modifying factor of this mutation, MRPS12 residue 68, has been found polymorphic in humans. Interestingly, we have found that CIs of human MRPS12 variant positions are higher than those of variant positions between human and Cercopithecidae. This suggests that these human variants alter function and are negatively selected, and only variants with lower CI (i.e., with lower phenotypic effect) survive. Therefore, amino acid substitutions in MRPS12 or other mitochondrial ribosomal proteins are interesting candidates to explain the incomplete penetrance of pathologic mt-rRNA mutations.

The expansion of sequence and molecular structure databases will undoubtedly facilitate the use of non-genetically manipulated animal models harboring CPD to unmask modifying factors for many homoplasmic mtDNA mutants.

## Author note

During the review of this manuscript, one of the referees made us note that an MRPS12 sequence from the Colobinae *Rhinopithecus roxellana* had been recently released in GenBank. The presence of an arginine at MRPS12 position 68 from this Colobinae species strongly supports that leucine 68 was fixed in the Papionini tribe after its divergence from the Cercopithecini tribe.

### Conflict of interest statement

The authors declare that the research was conducted in the absence of any commercial or financial relationships that could be construed as a potential conflict of interest.
