# Supplementary material for: An MRPS12 mutation modifies aminoglycoside sensitivity caused by 12S rRNA mutations
Source: Front Genet. 2015 Jan 14;5:469. doi: 10.3389/fgene.2014.00469 (PMC4294204; doi:10.3389/fgene.2014.00469)
Supplement: Supplementary file 1 [file DataSheet1.DOC]

Note 1. Mammalians with m.1494T as the wild-type allele. Common name is written in red color.

- Mammalia; Theria; Metatheria; Diprotodontia; Phascolarctidae; Phascolarctos; *Phascolarctos cinereus* (Koala).

- Mammalia; Theria; Metatheria; Notoryctemorphia; Notoryctidae; Notoryctes; *Notoryctes typhlops* (Marsupial mole).

- Mammalia; Theria; Eutheria; Afrotheria; Proboscidea; Elephantidae; Elephas; *Elephas maximus* (Asiatic elephant).

- Mammalia; Theria; Eutheria; Afrotheria; Proboscidea; Elephantidae; Loxodonta; *Loxodonta africana* (African savanna elephant), *Loxodonta cyclotis* (African forest elephant).

- Mammalia; Theria; Eutheria; Afrotheria; Proboscidea; Elephantidae; Mammut; *Mamut americanum* (American mastodon).

- Mammalia; Theria; Eutheria; Afrotheria; Proboscidea; Elephantidae; Mammuthus; *Mamuthus columbi* (Columbian mammoth), *Mamuthus primigenius* (Woolly mammoth).

- Mammalia; Theria; Eutheria; Euarchontoglires; Glires; Rodentia; Sciurognathi; Muroidea; Spalacidae; Myospalacinae; Eospalax; *Eospalax baileyi* (Plaetau zokor), *Eospalax cansus* (Gansu zokor), *Eospalax rothschildi* (Rothschild’s zokor).

- Mammalia; Theria; Eutheria; Euarchontoglires; Glires; Rodentia; Sciurognathi; Muroidea; Spalacidae; Rhyzomyinae; Rhizomys; *Rhizomys pruinosus* (Hoary bamboo rat):

- Mammalia; Theria; Eutheria; Euarchontoglires; Glires; Rodentia; Sciurognathi; Muroidea; Spalacidae; Spalacinae; Nannospalax; *Nannospalax ehrenbergi* (Middle East blind mole-rat), *Nannospalax galili* (Upper Galilee Mountains blind mole-rat), *Nannospalax golani* (Golan Heights blind mole-rat), *Nannospalax judaei* (Judean Mountains blind mole-rat).

- Mammalia; Theria; Eutheria; Euarchontoglires; Glires; Rodentia; Sciurognathi; Muroidea; Spalacidae; Spalacinae; Spalax; *Spalax carmeli* (Carmel blind mole-rat).

- Mammalia; Theria; Eutheria; Euarchontoglires; Primates; Haplorrhini; Simiiformes; Catarrhini; Cercopithecoidea; Cercopithecidae; Cercopithecinae; Allenopithecus; *Allenopithecus nigroviridis* (Allen’s swamp monkey).

- Mammalia; Theria; Eutheria; Euarchontoglires; Primates; Haplorrhini; Simiiformes; Catarrhini; Cercopithecoidea; Cercopithecidae; Cercopithecinae; Cercocebus; *Cercocebus chrysogaster* (golden-bellied mangabey), *Cercocebus torquatus* (Collared mangabey).

- Mammalia; Theria; Eutheria; Euarchontoglires; Primates; Haplorrhini; Simiiformes; Catarrhini; Cercopithecoidea; Cercopithecidae; Cercopithecinae; Cercopithecus; *Cercopithecus albogularis* (Sykes’ monkey), *Cercopithecus diana* (Diana monkey), *Cercopithecus lhoesti* (L’Hoest’s monkey), *Cercopithecus mitis* (Blue monkey).

- Mammalia; Theria; Eutheria; Euarchontoglires; Primates; Haplorrhini; Simiiformes; Catarrhini; Cercopithecoidea; Cercopithecidae; Cercopithecinae; Chlorocebus; *Chlorocebus aethiops* (African green monkey), *Chlorocebus pygerythrus* (Vervet), *Chlorocebus sabaeus* (Green Monkey), *Chlorocebus tantalus* (Tantalus).

- Mammalia; Theria; Eutheria; Euarchontoglires; Primates; Haplorrhini; Simiiformes; Catarrhini; Cercopithecoidea; Cercopithecidae; Cercopithecinae; Erythrocebus; *Erythrocebus patas* (Patas monkey).

- Mammalia; Theria; Eutheria; Euarchontoglires; Primates; Haplorrhini; Simiiformes; Catarrhini; Cercopithecoidea; Cercopithecidae; Cercopithecinae; Lophocebus; *Lophocebus aterrimus* (Black crested mangabey).

- Mammalia; Theria; Eutheria; Euarchontoglires; Primates; Haplorrhini; Simiiformes; Catarrhini; Cercopithecoidea; Cercopithecidae; Cercopithecinae; Macaca; *Macaca assamensis* (Assam macaque), *Macaca fascicularis* (Crab-eating macaque), *Macaca mulatta* (Rhesus monkey), *Macaca sylvanus* (Barbary ape), *Macaca thibetana* (Pere David's macaque).

- Mammalia; Theria; Eutheria; Euarchontoglires; Primates; Haplorrhini; Simiiformes; Catarrhini; Cercopithecoidea; Cercopithecidae; Cercopithecinae; Mandrillus; *Mandrillus sphinx* (Mandrill).

- Mammalia; Theria; Eutheria; Euarchontoglires; Primates; Haplorrhini; Simiiformes; Catarrhini; Cercopithecoidea; Cercopithecidae; Cercopithecinae; Papio; *Papio anubis* (Olive baboon), *Papio cynocephalus* (Yellow baboon), *Papio hamadryas* (Hamadryas baboon), *Papio kindae* (Kinda baboon), *Papio papio* (Guinea baboon).

- Mammalia; Theria; Eutheria; Euarchontoglires; Primates; Haplorrhini; Simiiformes; Catarrhini; Cercopithecoidea; Cercopithecidae; Cercopithecinae; Theropithecus; *Theropithecus gelada* (Gelada baboon).

- Mammalia; Theria; Eutheria; Euarchontoglires; Primates; Haplorrhini; Simiiformes; Catarrhini; Cercopithecoidea; Cercopithecidae; Colobinae; Colobus; *Colobus guereza* (Guereza).

- Mammalia; Theria; Eutheria; Euarchontoglires; Primates; Haplorrhini; Simiiformes; Catarrhini; Cercopithecoidea; Cercopithecidae; Colobinae; Nasalis; *Nasalis larvatus* (Proboscis monkey).

- Mammalia; Theria; Eutheria; Euarchontoglires; Primates; Haplorrhini; Simiiformes; Catarrhini; Cercopithecoidea; Cercopithecidae; Colobinae; Presbytis; *Presbytis melalophos* (Mitred leaf monkey).

- Mammalia; Theria; Eutheria; Euarchontoglires; Primates; Haplorrhini; Simiiformes; Catarrhini; Cercopithecoidea; Cercopithecidae; Colobinae; Procolobus; *Procolobus badius* (Western Red Colobus), *Procolobus verus* (Olive colobus).

- Mammalia; Theria; Eutheria; Euarchontoglires; Primates; Haplorrhini; Simiiformes; Catarrhini; Cercopithecoidea; Cercopithecidae; Colobinae; Pygathrix; *Pygathrix cinerea* (Gray shanked douc langur), *Pygathrix nemaeus* (Red shanked douc langur), *Pygathrix nigripes* (Black shanked douc), *Pygathrix roxellana* (Golden snub-nosed monkey).

- Mammalia; Theria; Eutheria; Euarchontoglires; Primates; Haplorrhini; Simiiformes; Catarrhini; Cercopithecoidea; Cercopithecidae; Colobinae; Rhinopithecus; *Rhinopithecus avunculus* (Tonkin snub-nosed monkey), *Rhinopithecus* brelichi (Grey snub-nosed monkey), *Rhinopithecus* strykeri (Myanmar snub-nosed monkey).

- Mammalia; Theria; Eutheria; Euarchontoglires; Primates; Haplorrhini; Simiiformes; Catarrhini; Cercopithecoidea; Cercopithecidae; Colobinae; Semnopithecus; *Semnopithecus entellus* (Hanuman langur).

- Mammalia; Theria; Eutheria; Euarchontoglires; Primates; Haplorrhini; Simiiformes; Catarrhini; Cercopithecoidea; Cercopithecidae; Colobinae; Simias; *Simias* *concolor* (pig-tailed langur).

- Mammalia; Theria; Eutheria; Euarchontoglires; Primates; Haplorrhini; Simiiformes; Catarrhini; Cercopithecoidea; Cercopithecidae; Colobinae; Trachypithecus; *Trachypithecus cristatus* (Silvery lutung), *Trachypithecus francoisi* (François' langur), *Trachypithecus germaini* (Indochinese lutung), *Trachypithecus hatinhensis* (Hatinh langur), *Trachypithecus johnii* (Nilgiri langur), *Trachypithecus obscurus* (Dusky leaf-monkey), *Trachypithecus shortridgei* (Shortridge’s langur), *Trachypithecus vetulus* (Purple faced langur).

Note 2. List of 90 species of the Cercopithecidae family analyzed for m.1494 position. All these species, except *Papio ursinus*, have m.1494T. This position is apparently polymorphic in *Papio ursinus* (m.1494C in NC_020010.2 and JX946205.2 and m.1494T in JX946204.2).

**Subfamily Cercopithecinae**

**Tribe Cercopithecini:**

*Allenopithecus nigroviridis*, *Cercopithecus albogularis*, *Cercopithecus ascanius*, *Cercopithecus campbelli*, *Cercopithecus cephus*, *Cercopithecus denti*, *Cercopithecus diana*, *Cercopithecus doggetti*, *Cercopithecus dryas*, *Cercopithecus erythrogaster*, *Cercopithecus erythrotis*, *Cercopithecus hamlyni*, *Cercopithecus kandti*, *Cercopithecus lhoesti*, *Cercopithecus mitis*, *Cercopithecus mona*, *Cercopithecus neglectus*, *Cercopithecus nictitans*, *Cercopithecus petaurista*, *Cercopithecus pogonias*, *Cercopithecus preusii*, *Cercopithecus roloway*, *Cercopithecus solatus*, *Cercopithecus wolfi*, *Chlorocebus aethiops*, *Chlorocebus cynosuros*, *Chlorecebus pygerythrus*, *Chlorebus sabaeus*, *Chlorocebus tantalus*, *Erythrocebus patas*, *Miopithecus ogouensis*, *Miopithecus talapoin*.

**Tribe Papionini:**

*Cercocebus atys*, *Cercocebus chrysogaster*, *Cercocebus torquatus*, *Lophocebus albigena*, *Lophocebus aterrimus*, *Macaca* *arctoides*, *Macaca assamensis*, *Macaca brunnescens*, *Macaca cyclopis*, *Macaca fascicularis*, *Macaca fuscata*, *Macaca hecki*, *Macaca maura*, *Macaca mulatta*, *Macaca nemestrina*, *Macaca nigra*, *Macaca nigrescens*, *Macaca ochreata*, *Macaca pagensis*, *Macaca radiata*, *Macaca silenus*, *Macaca sinica*, *Macaca sylvanus*, *Macaca thibetana*, *Macaca tonkeana*, *Mandrillus leucophaeus*, *Mandrillus sphinx*, *Papio anubis*, *Papio cynocephalus*, *Papio hamadryas*, *Papio kindae*, *Papio papio*, *Papio ursinus*, *Theropithecus gelada*.

**Subfamily Colobinae**

**Tribe Colobini:**

*Colobus guereza*, *Procolobus badius*, *Procolobus verus*.

**Tribe Presbytini:**

*Nasalis larvatus*, *Presbytis melalophos*, *Pygathrix cinerea*, *Pygathrix nemaeus*, *Pygathrix nigripes*, *Rhinopithecus* *avunculus*, *Rhinopithecus bieti*, *Rhinopithecus brelichi*,

*Rhinopithecus* *roxellana*, *Rhinopithecus strykeri*, *Semnopithecus entellus*, *Simias concolor*, *Trachypithecus cristatus*, *Trachypithecus francoisi*, *Trachypithecus germaini*, *Trachypithecus hatinhensis*, *Trachypithecus johnii*, *Trachypithecus obscurus*, *Trachypithecus pileatus*, *Trachypithecus shortridgei*, *Trachypitechus vetulus*.

Note 3. List of 70 mammalian species included in the CI analyses of the MRPS12 protein.

Eukaryota, Metazoa, Chordata, Craniata, Vertebrata, Euteleostomi, Mammalia,

Eutheria,

Afrotheria,

Chrysochloridae, Chrysochlorinae, Chrysochloris, *Chrysochloris asiatica*

Hyracoidea, Procaviidae, Procavia, *Procavia capensis*

Macroscelidea, Macroscelididae, Elephantulus, *Elephantulus edwardii*

Proboscidea, Elephantidae, Loxodonta, *Loxodonta africana*

Sirenia, Trichechidae, Trichechus, *Trichechus manatus latirostris*

Tenrecidae, Tenrecinae, Echinops, *Echinops telfairi*

Tubulidentata, Orycteropodidae, Orycteropus, *Orycteropus afer afer*

Euarchontoglires,

Glires,

Lagomorpha,

Ochotonidae, Ochotona, *Ochotona princeps*

Leporidae, Oryctolagus, *Oryctolagus cuniculus*

Rodentia,

Hystricognathi,

Bathyergidae, Heterocephalus, *Heterocephalus glaber*

Caviidae, Cavia, *Cavia porcellus*

Chinchillidae, Chinchilla, *Chinchilla lanigera*

Octodontidae, Octodon, *Octodon degus*

Sciurognathi,

Dipodidae, Dipodinae, Jaculus, *Jaculus jaculus*

Heteromyidae, Dipodomyinae, Dipodomys, *Dipodomys ordii*

Muroidea,

Cricetidae,

Arvicolinae, Microtus, *Microtus ochrogaster*

Cricetinae, Cricetulus, *Cricetulus griseus*

Neotominae, Peromyscus, *Peromyscus maniculatus bairdii*

Muridae,

Murinae, Mus, *Mus musculus*, Rattus, *Rattus norvegicus*

Sciuridae,

Xerinae, Marmotini, Ictidomys, *Ictidomys tridecemlineatus, Spermophilus tridecemlineatus*

Primates,

Haplorrhini,

Catarrhini,

Cercopithecidae, Cercopithecinae, Chlorocebus, *Chlorocebus sabaeus,* Macaca, *Macaca fascicularis*, *Macaca mulatta*, Papio, *Papio Anubis*

Hominidae, Gorilla, *Gorilla gorilla gorilla*, Homo, *Homo sapiens*, Pan, *Pan paniscus*, *Pan troglodytes*, Pongo, *Pongo abelii*

Platyrrhini, Cebidae, Saimiriinae, Saimiri, *Saimiri boliviensis boliviensis*

Tarsiiformes, Tarsiidae, Tarsius, *Tarsius syrichta*

Strepsirrhini, Lemuriformes, Cheirogaleidae, Microcebus, *Microcebus murinus*

Scandentia, Tupaiidae, Tupaia, *Tupaia* *belangerii*, *Tupaia chinensis*

Laurasiatheria,

Carnivora,

Caniformia,

Canidae, Canis, *Canis lupus familiaris*

Mustelidae, Mustelinae, Mustela, *Mustela putorius furo*

Odobenidae, Odobenus, *Odobenus rosmarus divergens*

Phocidae, Leptonychotes, *Leptonychotes weddellii*

Ursidae, Ailuropoda, *Ailuropoda melanoleuca*

Feliformia, Felidae,

Felinae, Felis, *Felis catus*

Pantherinae, Panthera, *Panthera tigris altaica*

Cetartiodactyla,

Cetacea,

Mysticeti, Balaenopteridae, Balaenoptera, *Balaenoptera acutorostrata scammoni*

Odontoceti,

Delphinidae, Orcinus, *Orcinus orca*, Tursiops, *Tursiops truncatus*

Lipotidae, Lipotes, *Lipotes vexillifer*

Physeteridae, Physeter, *Physeter catodon*

Ruminantia, Pecora, Bovidae,

Antilopinae, Pantholops, *Pantholops hodgsonii*

Bovinae, Bos, *Bos mutus*, *Bos taurus*, Bubalus, *Bubalus bubalis*

Caprinae, Capra, *Capra hircus*, Ovis, *Ovis aries*

Suina, Suidae, Sus, *Sus scrofa*

Tylopoda, Camelidae, Camelus, *Camelus ferus*, Vicugna, *Vicugna pacos*

Chiroptera,

Megachiroptera, Pteropodidae, Pteropodinae, Pteropus, *Pteropus alecto*, *Pteropus vampyrus*

Microchiroptera, Vespertilionidae, Eptesicus, *Eptesicus fuscus*, Myotis, *Myotis* *brandtii*, *Myotis davidii*, *Myotis lucifugus*

Insectivora,

Erinaceidae, Erinaceinae, Erinaceus, *Erinaceus europaeus*

Soricidae, Soricinae, Sorex, *Sorex araneus*

Talpidae, Condylura, *Condylura cristata*

Perissodactyla,

Equidae, Equus, *Equus caballus*

Rhinocerotidae, Ceratotherium, *Ceratotherium simum simum*

Xenarthra, Cingulata, Dasypodidae, Dasypus, *Dasypus novemcinctus*

Metatheria, Didelphimorphia, Didelphidae, Monodelphis, *Monodelphis domestica*
